# Consolidation of Fir Wood by Poly(vinyl butyral-*co*-vinyl alcohol-*co*-vinyl acetate) Treatment: Study of Surface and Mechanical Characteristics

**DOI:** 10.3390/polym12051039

**Published:** 2020-05-02

**Authors:** Danial Harandi, Javier González-Benito, Dania Olmos

**Affiliations:** Dpto. Ciencia e Ingeniería de Materiales e Ingeniería Química, IQMAAB, Universidad Carlos III de Madrid Avda. Universidad, 15, Leganés, 28911 Madrid, Spain; dharandi@pa.uc3m.es (D.H.); javid@ing.uc3m.es (J.G.-B.)

**Keywords:** consolidation, Fir wood, poly(vinyl butyral-*co*-vinyl alcohol-*co*-vinyl acetate), surface characterization, mechanical characterization

## Abstract

The ability of poly(vinyl butyral-*co*-vinyl alcohol-*co*-vinyl acetate) (PVBVA) to consolidate Fir wood was studied in terms of the surface and mechanical properties’ changes. Two variables were considered to treat the wood: (i) the concentration (5 and 10 wt.%) of PVBVA solutions and (ii) the method of application (brushing and immersion). The presence of PVBVA on the wood surfaces was confirmed by infrared spectroscopy. Surface roughness measured by optical profilometry did not reveal changes in the topography of the samples, and appropriate visual appearance was confirmed. Contact angle measurements showed that a droplet of the 10%-PVBVA solution needed ~50 s to reach the same contact angle decreasing rate as that measured for the 5%-PVBVA solution, suggesting there was some kind of induction time till the spreading process was no longer controlled by the viscosity, but by the solution-wood interactions. Water contact angle (WCA) measurements proved a more hydrophobic surface of the PVBVA-treated samples, compared to untreated wood. Mechanical characterization of the samples was done macroscopically by a three-point bending test and locally by the Shore D and Martens hardness (MH). Only results from MH experiments provided comparative results, indicating that treatment with PVBVA solutions increased wood hardness locally, being enhanced with solution concentration. The best surface mechanical properties were obtained for the samples immersed in the 10%-PVBVA solution.

## 1. Introduction

Wood was one of the earliest and is the most frequent material present in buildings, furniture, and decorative objects. However, wood-based materials, as a natural biodegradable product, lose their aesthetic and mechanical properties due to different aging phenomena. For this reason, conservation and restoration of wood structural elements is an important issue to address by experts in the field of the authenticity preservation of artwork for instance. Conservation and restoration of artworks implies the use of different strategies for the preservation of different kinds of objects, such as paintings, sculptures, decorative objects, furniture, and other objects that have lost their functionality, their aesthetic, and/or mechanical properties by human use or by the effects of time [1,2,3]. However, all these strategies imply processes that may be difficult, time consuming, or not effective enough. Therefore, restoration scientists and conservators try to find new, more effective, and easier methods and techniques to preserve or restore damaged artworks.

One of the most common approaches to consolidate degraded wood consists of the application of products that can penetrate into the cell walls of the wood or in the voids at the surface to improve its mechanical strength [1]. The efficiency of the treatment is usually associated with the nature of the product used, its penetration in the wood, and its permanence over the time [4]. However, there may be other factors affecting the final performance of certain treatments, for instance: (i) the method used to apply the product (immersion, brushing, etc.); (ii) the nature of the substrate (the wood); (iii) the use of particular pre-treatments or post-treatments on the wood or the consolidant; and (iv) in service conditions. 

The general purpose of wood consolidation treatments is to ensure better internal structural cohesion and improved mechanical strength. Usually, there is a need to fill in, at least partially, the voids or wood cells to restore the mechanical properties lost [1,2,3]. In the consolidation of historical wood objects, also surface properties and appearance are important. That is why surface brightness or surface mechanical properties, among others, should be investigated.

Polymers have been extensively used on wooden surfaces [1,5,6], including epoxy and polyester resins [5], polyurethanes [7], cellulose derivatives [8], and a wide range of thermoplastic polymers [9,10]. Within these, two of the most widely used due to their good optical properties are acrylate-based polymers, such as Paraloid B72 [11,12,13,14,15], and those derived from polyvinyl butyral (PVB) [16,17]. Commercial PVB contains 17–22 wt.% of vinyl alcohol, 1–3 wt.% of vinyl acetate, and 75–82 wt.% of vinyl butyral units [18]. In this work, poly(vinyl butyral-*co*-vinyl alcohol-*co*-vinyl acetate) (PVBVA) was chosen for surface and deep modification of Fir wood due to is good optical properties, high adhesion strength, and high ductility.

Apart from the selection of the consolidating agent and the study with common characterization methods, to better understand the consolidation mechanism, it would be interesting to provide a new insight into new characterization alternatives to get other information not available up to now. For example, one of the major challenges is to understand better the penetration process of the consolidating agent into the wood cells and, more specifically, how it can affect the final properties of the restored material. For example, how can the absorption of the polymer solution affect the final properties of the material? Does the rate of absorption affect the physico-chemical and mechanical properties of the material? Possibly, a novel characterization study trying to answer these questions may be to monitor dynamic solution up-take due to adsorption and capillarity. In this work, the measurement of the contact angle as a function of time is proposed as a new approach to study the rate of penetration of the polymer solution into untreated wood specimens. 

Surface characterization of the materials is another important issue to be addressed [19,20,21,22]. Surface properties, such as roughness, may play an important role in the effectiveness of surface treatments on wood [23]. For that purpose, the study of surface roughness before and after wood treatment is proposed. The second important issue is mechanical behavior [24,25,26,27,28,29]. Typically, the overall mechanical properties of the materials are evaluated. For instance, three-point bending tests help us to study the mechanical behavior of the materials as a whole. However, obtaining the information of local mechanical properties is also very important. For this reason, universal hardness measurements (Martens hardness) are proposed to investigate the local mechanical response of the materials exactly on the surface of the wood, close to the area of influence of the treated surfaces. 

In this context, the aim of this work is to study the surface and mechanical properties of Fir wood surfaces treated with poly(vinyl butyral-*co*-vinyl alcohol-*co*-vinyl acetate) (PVBVA). The concentration (5% or 10%, wt.%) and method of application (brushing or immersion) of the PVBVA solutions are the variables selected for this study. Contact angle monitoring of the polymer solution on the surface of wood is done to study the effect of polymer concentration on the absorption rate and solution penetration. In-depth surface analysis is done using surface roughness measurements, water contact angle, and local mechanical properties (Martens hardness (MH)). Complementary, three-point bending test experiments are done to characterize the global mechanical properties of the materials and correlate them with the local mechanical response (MH) and surface characteristics in order to probe the effectiveness of the different surface treatments on the consolidation of wooden artefacts. 

## 2. Materials and Methods 

### 2.1. Materials

Softwood European silver Fir wood (*Abies alba*) was chosen. In previous studies [4], this type of wood has been used for evaluating the effects of the consolidations and in the construction of historical wood artefacts.

**Figure 1:**
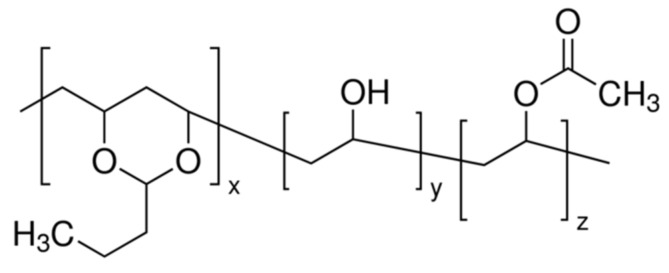
Chemical structure of poly(vinyl butyral-co-vinyl alcohol-co-vinyl acetate) (PVBVA) (from Sigma-Aldrich catalog, Reference 182567).

Poly (vinyl butyral-*co*-vinyl alcohol-*co*-vinyl acetate) (PVBVA), with CAS Number 27360-07-2, average molecular weight M_w_ = 50,000–80,000 g·mol^−1^, average content of vinyl butyral of 80 wt. %, and T_g_ ~ 62–72 °C, was used. The polymer was supplied by Sigma-Aldrich (Madrid, Spain, Reference Number 182567). The chemical structure of PVBVA is shown in Figure 1. 

Ethanol (Sigma-Aldrich, St. Luis, MO, USA, absolute for HPLC, ≥ 99.8%) and acetone (Sigma-Aldrich, St. Luis, MO, USA, HPLC Plus, for HPLC, GC, and residue analysis, 99.9% grade) were used to prepare the PVBVA solutions. Mixtures of 1:1 ethanol:acetone were used to prepare the PVBVA solutions. Distilled and de-ionized water was used for contact angle measurements. 

### 2.2. Sample Preparation

After cutting wood samples, surfaces were sanded with H 240 grit size sandpaper and conditioned in a climate chamber at a temperature of T = 20 ± 2 °C and relative humidity RH = 67 ± 5% until constant weight (reaching a water content at equilibrium of ~12% by weight). Before applying PVBVA solutions, any possible dust remaining was removed from the wood surfaces by compressed air blowing. To apply the PVBVA solution on the wood surfaces, two methods were used, brushing with a soft brush and specimen immersion in the solution for 24 h. Brushing was chosen due to its wide use in indoor and outdoor consolidation historical wooden objects. It is easy to use on large specimens for which other methods cannot be applied, such as saturation and immersion. Immersion was selected in order to evaluate if the method used was critical in terms of the final consolidant penetration. For each method, two concentrations of polymer solution were considered, 5% and 10% by weight. Specimens were kept at room temperature for one week to allow solvent evaporation, and they were finally conditioned in the climate chamber for one month at T = 20 ± 2 °C and RH = 67 ± 5% until constant weight.

### 2.3. Equipment

The structure of PVBVA coatings was studied by attenuated total reflection Fourier transformed infrared spectroscopy (ATR-FTIR), using a Shimadzu IRAffinity-1S spectrometer equipped with a Golden Gate ATR accessory (diamond window), collecting the spectra at room temperature from 600 to 4000 cm^−1^ with a resolution of 4 cm^−1^ and averaging 32 scans. OMNIC 6.0 software (ThermoFisher Scientific Inc., Waltham, MA USA) was used to analyze the spectra. 

To investigate the solution uptake and specific interactions between the solutions and the Fir wood, contact angle measurements were carried out by the sessile drop method using an OCA 15 Plus instrument from KRÜSS GmbH (Hamburg, Germany). To perform the dynamic solution up-take experiments, a 5 µL drop of solution was deposited at 1 µL·s^−1^ on the wood surfaces (specimens of 10 × 10 × 15 mm^3^), and contact angles were measured at 2, 20, 40, 60, 80, 100, 120, 140, 160 and 190 s after depositing the droplet. SCA20 software was used for image analyses. A small container with the solvent (1:1 ethanol/acetone) was placed in the sample chamber to saturate the atmosphere and reduce solvent evaporation from the solutions. Wettability was also studied by measuring the contact angles formed by water drops also with the sessile drop method and similar conditions (5 µL at 1 µL·s^−1^). Contact angles were measured 2 s after depositing the droplet [30]. The final contact angle value was obtained from the average of at least 20 measurements per sample. 

To study the surface topography of wood, optical profilometry was used (OLYMPUS DSX500, Olympus Iberia, Barcelona, Spain). One specimen of each sample with dimensions of 2 × 1 × 5 mm^3^ was analyzed before and after each surface treatment. Surface roughness data were obtained after analyzing the topography of the samples according to standard UNE-EN ISO 4288. Two roughness parameters were selected for this study, R_a_ (arithmetical mean roughness) and S_a_ (arithmetical mean height). To calculate R_a_ and S_a_, the cut-off was estimated using the average value of the parameter RSm obtained from the analysis of 10 linear profiles (5 horizontal lines and 5 vertical lines). The parameter RSm, the mean width of the profile elements, indicates the average value of the length of the profile element, Xs along the sampling length, S_l_ [31]. The cut-off wavelength (λ_c_) was estimated considering that the evaluation length should include at least 5 sampling lengths, i.e., λ_c_ equals 5 times the average value of the mean width, RSm. This criterion was chosen according to ISO 4288 [32]. In order to ensure the inspection of the same part of wood before and after the treatments, a specific region of the specimen was marked and photographed [33,34,35]. A more detailed description of the definition of different roughness parameters can be found in [31].

**Figure 2:**
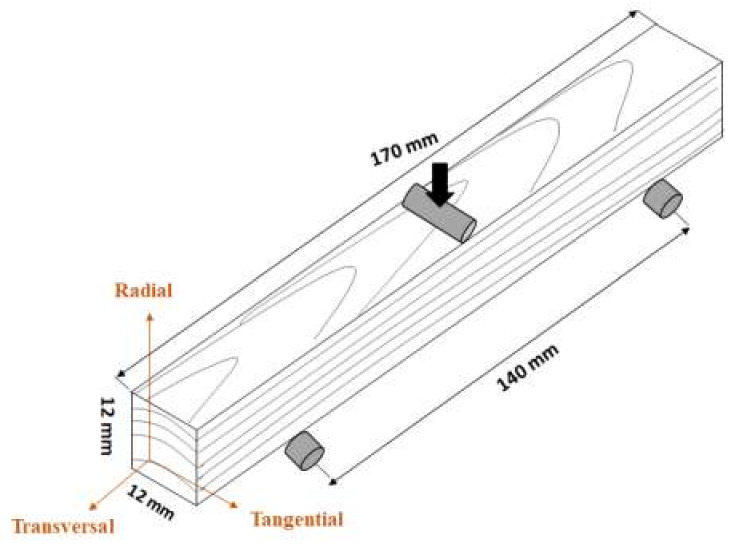
Scheme showing the wood specimens dimensions, and assembly positions for three-point bending test measurements (adapted from [37]).

The mechanical properties of wood materials were studied by performing three-point bending tests. The experiments were done according to ASTM D 1037 [36] in a universal testing machine (MICROTEST EM2/200, Madrid, Spain) with a load cell of 10 kN. Specimens with dimensions of 12 × 12 × 170 mm^3^ were tested setting the span at 140 mm, applying the load at a rate of 6 mm/min (Figure 2). Five specimens were tested for each sample. The modulus of rupture (MOR) and modulus of elasticity (MOE) were calculated according to the following equations:

(1) $$\mathrm{MOR}\;\left(\mathrm{MPa}\right)=\frac{3\mathrm{PL}}{2{\mathrm{bd}}^{2}}$$

(2) $$\mathrm{MOE}\;\left(\mathrm{MPa}\right)=\frac{P_{1}L^{3}}{4{\mathrm{bd}}^{3}y_{1}}$$

where P is the maximum load (N), determined experimentally from the curves, L is the length of the span (mm), which was set at 140 mm [36], b, is the width of the specimen (mm), d is the thickness of the specimen (mm), P_1_ is the load at the proportional limit (N), and y_1_ is the center deflection (mm) at the proportional limit load, which was set at approximately 40% of the maximum load [37,38]. 

The surface hardness of the samples was obtained from Shore D hardness tests. Hardness data were taken from forty replicas in the radial and tangential direction and ten replicas in the longitudinal direction for each wood specimen using a J. BOT S.A. Shore D hardness tester. Universal hardness (MH) measurements were done using a Zwick Z 2.5 hardness testing machine (Zwick GmbH & Co., Ulm, Germany). Five indentations were made on each specimen. The loading and unloading speed was 3 mm/min waiting for 2 s of relaxation time between loading and unloading.

## 3. Results

### 3.1. Structural Characterization

**Figure 3:**
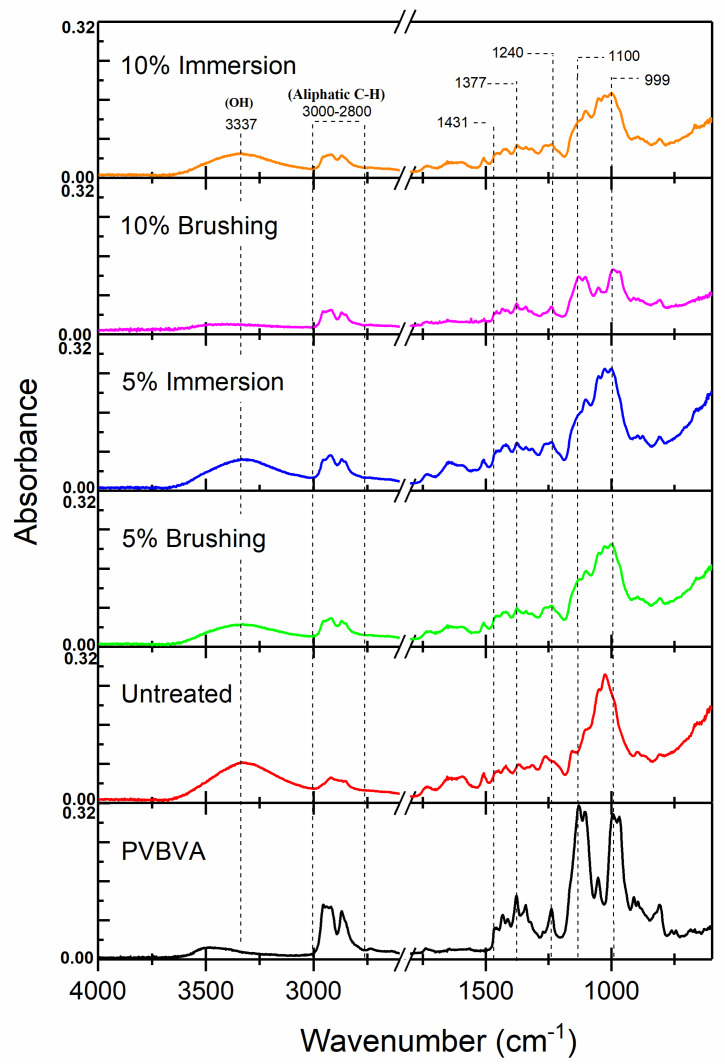
ATR-FTIR spectra of wood samples under study: untreated, 5%-brushing, 10%-brushing, 5%-immersion, and 10%-immersion. Spectra of PVBVA are included for reference.

In Figure 3, the ATR-FTIR spectra of the samples under study are shown. As a reference, the spectrum of a casted film of PVBVA was also collected. In Table 1, assignments of the main absorption bands of PVBVA are presented. The characteristic broad band centered at 3486 cm^−1^ was assigned to the stretching vibration of the –OH group (the appearance of a broad band is usually explained by the presence of hydrogen bonds or OH with different surroundings). The antisymmetric C–H stretching vibrations of methyl, ν_as_(CH_3_), and methylene, ν_as_(CH_2_), groups appeared close to 2950 cm^−1^ and 2926 cm^−1^, respectively, which in the treated wood surfaces overlapped and probably corresponded to the shoulder observed at 2953 cm^−1^. The corresponding symmetric C–H stretching vibrations for CH_3_ and CH_2_ were combined in the band centered at 2870 cm^−1^. Antisymmetric bending vibration of CH_3_ at ~1460 cm^−1^ usually overlapped with CH_2_ scissoring at 1433 cm^−1^. The peak centered at 1377 cm^−1^ could be assigned to the symmetric bending of CH_3_ and the in-plane deformation of C–OH [39]. The absorption band of stretching associated with the vibration of the ester group (O–C(O)–C) appeared at 1240 cm^−1^, while the absorption C−O stretching, coupled to adjacent C−C vibration, appeared at 1055 cm^−1^. 

As can be observed in Figure 3, either the untreated and treated specimens presented a strong broad band corresponding to the O–H stretching at 3300–4000 cm^−1^ [45]. In treated samples, the absorbance of this band decreased, compared to that of untreated wood, evidencing the surface treatment with the polymer since the –OH content of PVBVA (Figure 1) was quite lower than the structural components of Fir wood [46]. The presence of PVBVA polymer was also proven comparing the absorbance of the stretching vibrations of the alkyl group, ν_as_(CH_3_, CH_2_) and (ν_s_(CH_3_, CH_2_), in treated samples with the untreated one. The characteristic absorption bands of wood were in good agreement with those described in previous works [46,47,48]. 

The infrared spectra of the fingerprint region of wood samples, before and after treatments with PVBVA, were also compared. The peak at 1431 cm^−1^ was assigned to the CH_2_ bending vibration of PVBVA. The C–O–C and C–O stretching modes that appeared at 1100 cm^−1^ and 999 cm^−1^, respectively, could only be observed after treatments with PVBVA. The absorbance band at 1268 cm^−1^ was reduced in the treated samples, as well. Besides, it seemed that this effect was enhanced when the method used was brushing and when the concentration of the PVBVA solutions increased from 5% to 10%. 

From the FTIR results, one would infer that the higher the concentration of the PVBVA solution, the thicker the coating, and when the treatment method was brushing, thicker polymeric coatings were also obtained, at least when solutions of 5% and 10% of PVBVA were used. The band at 1240 cm^−1^ associated with the vibration mode of the acetate group [9,10] also appeared after the treatment, as expected, taking into account that this group formed part of the PVBVA structure (Figure 1). Finally, in the modified samples, the absorption band at 1377 cm^−1^, which corresponded to the CH_3_ bending mode of the PVBVA [44], was also observed, evidencing the presence of PVBVA after the surface treatments. 

### 3.2. Surface Characterization

**Figure 4:**
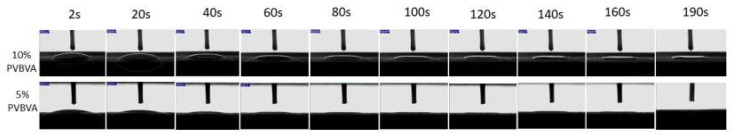
Evolution of PVBVA droplets’ profiles associated with the solution up-take process for 5% PVBVA (bottom) and for 10% PVBVA (top) solutions.

**Figure 5:**
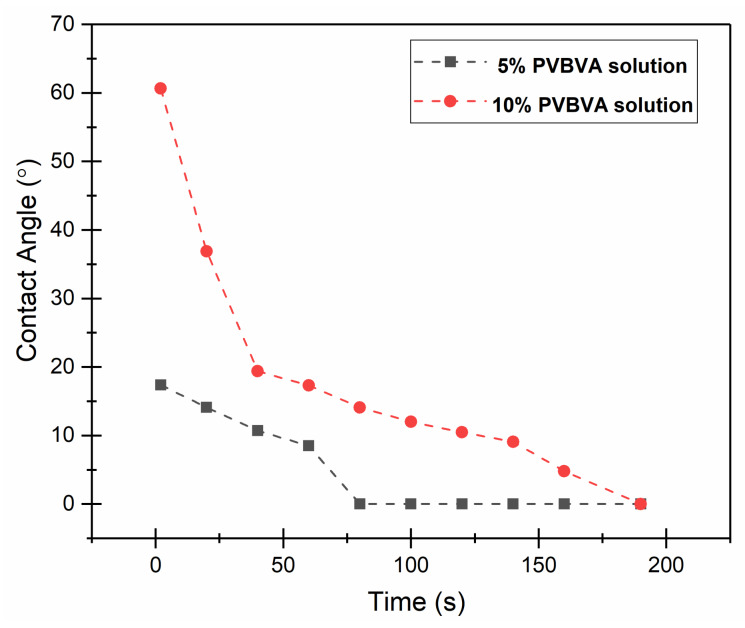
Contact angles of PVBVA solutions as a function of time for the 5% PVBVA (black) and 10% PVBVA (red) solutions.

In Figure 4 and Figure 5, the results of the solution up-take experiments are presented. As an example, in Figure 4 is shown the evolution over time of PVBVA solution drops on untreated wood surfaces. In Figure 5, the contact angles measured as a function of time are presented. Two main results can be extracted from these experiments: the contact angle at the beginning of the experiment and the total time required for the solution to be spread out in the wood surface or integrated in its structure (if the solution penetrated the wood). At a contact time of zero, the contact angle greatly depended on the concentration of the PVBVA solution, 17° for the 5% PVBVA solution and 61° for the 10% PVBVA solution, which might indicate weaker interactions between the most concentrated solution and the wood. However, at the beginning of the experiment, the fastest reduction of the solution contact angle occurred for the most concentrated solution, suggesting another possible explanation of the apparent higher contact angle, which was the highest viscosity of the 10% solution. Although contact angles were less than 90°, indicating a reasonably good solution adhesion on the wood surface, the ability of solution spreading to affect the quality of the final coating, and even the uniform product penetration compromising the treatment success with respect to the consolidation of wooden materials. 

On the other hand, it was observed that complete absorption of the solutions was attained after 60 s for the 5% PVBVA solution and 160 s for the 10% PVBVA solution. These results could be explained by considering the contribution of the viscosity. More diluted solutions should have lower viscosity, thus favoring spreading processes. In fact, the droplet of the 10% PVBVA solution needed a time of about 50 s to reach the same contact angle decreasing rate. Therefore, it could be considered that there was a kind of induction time when the spreading process was no longer controlled by the viscosity to be controlled by solution-wood interactions. 

Water contact angle (WCA) measurements were conducted to study the effect of the treatments on the surface properties of the wood. Untreated wood was used as the reference material. Figure S1 (right) shows, as examples, representative images of water droplets on the materials under study. On the other hand, in Figure S1 (left), average values of WCA are shown. A low water contact angle in untreated wood seemed reasonable due to the moisture content of untreated samples or the higher relative amount of hydroxyl groups as the FTIR spectra showed (Figure 3), suggesting a more hydrophilic surface. Upon the treatment with PVBVA solutions, the contact angle clearly increased regardless of the method of application or the concentration of the solution. For untreated wood, the contact angle was 32.5°, while for the PVBVA-coated samples, the WCA was found in the range of 86–90°, indicating that any treatment with PVBVA solutions greatly increased the hydrophobicity, which would be a good characteristic with respect to the prevention of water uptake of the wood in humid atmospheres, retarding degradation from weathering and avoiding volumetric changes of the samples. 

**Figure 6:**
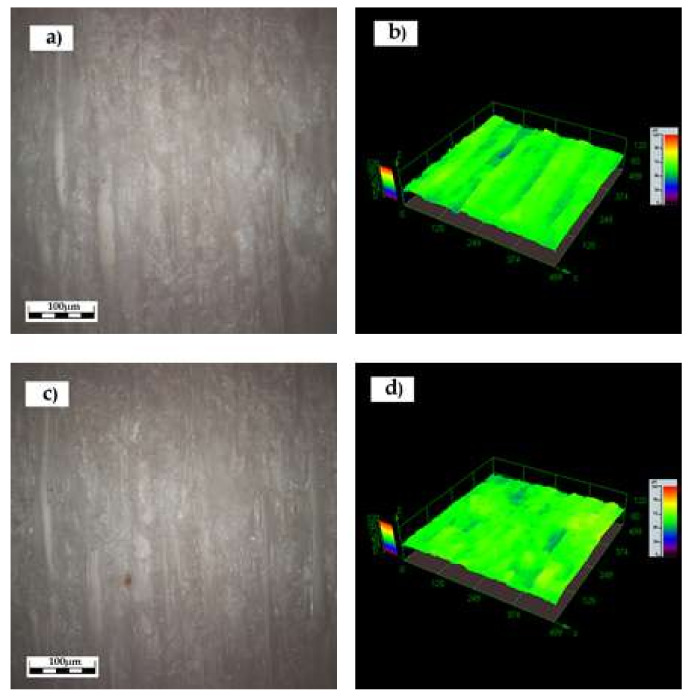
Optical profilometry images corresponding to the sample treated with 5%-immersion PVBVA. (**a**) and (**b**) show the 2D and 3D images of the sample before treatment and (**c**) and (**d**) the corresponding ones upon the treatment with the 5% PVBVA solution at 555×.

In Figure 6, topographical images obtained by optical profilometry are shown for the sample treated with the 5%-PVBVA solution after immersion treatment. Figure 6a,b corresponds to the 2D and 3D images of the neat surface of wood before the treatment with the polymer solution, whereas in Figure 6c,d, the corresponding images for the same sample after the application of the 5% PVBVA solution by immersion are shown. Similar images were obtained for the other samples: 5%-brushing; 10%-brushing, and 10%-immersion (see the Electronic supplementary material (Figure S1, Figure S2, and Figure S3, respectively)). The purpose of this characterization was to measure surface roughness before the application of the polymer, that is from Figure 6a,b, and after the application of the polymer, that is from Figure 6c,d, for the 5%-immersed sample. The same measurements were done for the other samples from the images shown in Figures S2, S3 and S4. 

Quantitative analysis was done to address the effect of surface treatment on the samples. In Table 2, the Ra (μm) and Sa (μm) of the samples under study are collected. In order to make a better comparison between the untreated wood and those treated, the values of the roughness parameters were obtained for the same region of the sample before and after the corresponding treatment (Table 2). Results indicated that surface roughness remained approximately constant after any of the surface treatments considered in this work. These results suggested that for consolidation wood by PVBVA, neither the method of application nor the concentration of the polymer solution affected surface roughness, at least at the scale considered here, using optical profilometry. In principle, these surface roughness results were in accordance with no evidence of the negative impact of the PVBVA treatments explored in this work on the visual appearance of the wood surface. 

**Figure 7:**
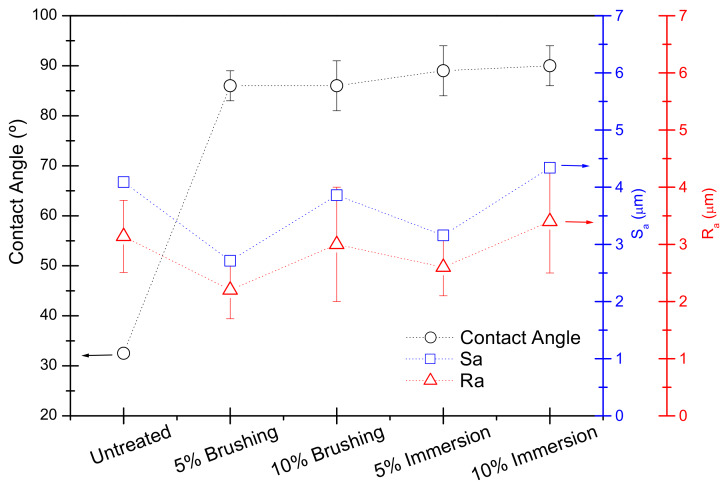
Comparative graph showing the variation of the contact angle and surface parameters for PVBVA-treated wood samples.

To complete the surface analysis, it was interesting to evaluate if there was any correlation between the surface parameters analyzed, contact angle data, and roughness parameters. In Figure 7, a comparative graph is given to illustrate this. Since the roughness parameters (Sa or Ra) remained approximately constant, roughness would not be one of the factors affecting surface wettability. Therefore, the changes in water contact angle (WCA) were mainly due to changes in the physico-chemical interactions between the wood surface and the liquid. This meant that changes in the hydrophobicity of the samples came from specific interactions and were not due to changes in surface roughness. 

### 3.3. Mechanical Characterization

**Figure 8:**
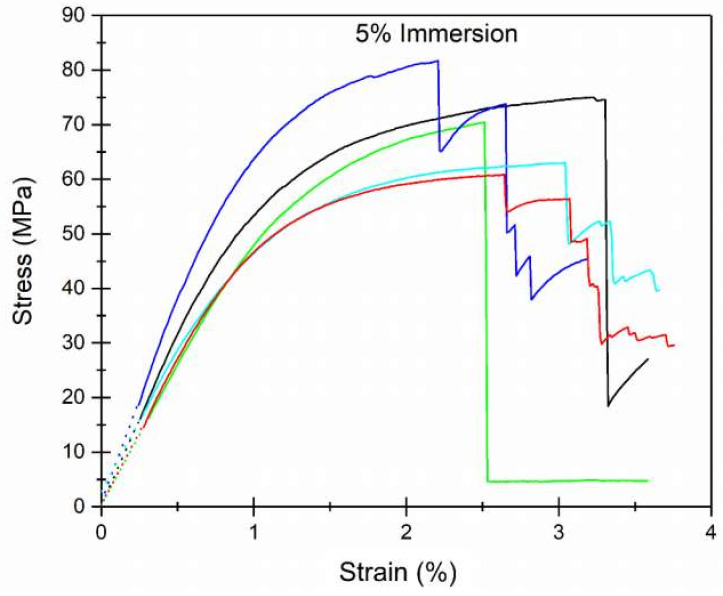
Stress (MPa) vs. strain (%) curves obtained by three-point bending tests for the sample of Fir wood immersed in a 5% PVBVA solution (note: see the Electronic supplementary material for other samples).

The macroscopic mechanical behavior was studied from three-point bending tests. As an example, in Figure 8, some stress (MPa) vs. strain (%) curves obtained from the three-point bending tests are presented, showing the typical stress-strain profiles of Fir wood. The original force (kN) vs. displacement (mm) curves and the stress (MPa) vs strain (%) curves obtained in the three-point bending test experiments for all the samples can be observed in Figures S5 and Figure S6, respectively. 

From the three-point bending tests, it was possible to calculate the MOR and MOE for all the samples under study (Table 3). The values obtained for the untreated Fir wood were in good agreement with others previously published [4]. On the other hand, the treatment with PVBVA solution seemed to improve slightly the mechanical resistance of the wood (Table 3). However, when comparing between treatment methods or solution concentration used in this work, a significant influence on the final macroscopic mechanical properties was not observed (Table 3). However, without considering the error of the measurements, it seemed that samples treated by immersion with a 10% solution of PVBVA had slightly higher mechanical properties. A possible explanation may be associated with the consolidation effect due to integration of the PVBVA within the wood microstructure, leading to a synergistic effect. In principle, it seemed that this polymer integration was dependent on two factors: (i) penetration and (ii) amount of polymer. In principle, the best wood consolidation would be attained when the highest solution penetration was achieved and when the required amount of polymer was available to fill the open microstructure of the wood completely. 

**Figure 9:**
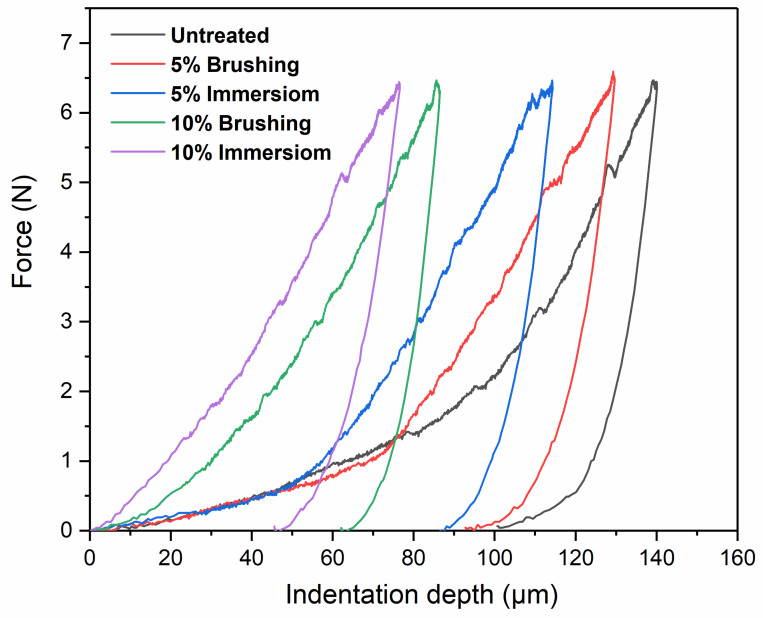
Representative force (N) vs. displacement (μm) curves obtained from Martens hardness measurements for the wood samples under study.

In Figure 9, typical indentation curves are shown for all the samples studied. For the same applied load, the indentation depths changed as a function of surface treatment. Increasing the concentration of the solution generally decreased the indentation depth. For example, for the samples treated with brushing, the indentation depth decreased 153 μm (untreated sample), 145 μm (5% PVBVA, brushing), and 115 μm (10% PVBVA, brushing). For the samples treated by immersion in the polymer solution, a similar trend was observed, obtaining indentation depths of 149 μm (5% PVBVA-immersion) and 82 μm (10% PVBVA-immersion). 

Comparing the method used, brushing vs. immersion, it was observed that when the concentration of the PVBVA solution was 5% (wt.%), practically no significant differences were found irrespective of the method of preparation. However, for solutions with higher concentrations (10% PVBVA), the indentation depth was found to be significantly lower for the sample prepared by immersion. These results point out that the concentration of the solution played an important role in the local mechanical properties. In fact, the effect of concentration was more noticeable in terms of the mechanical properties than the method of application. Again, it seemed that when the amount of polymer available increased, a higher capacity of filling voids existed, leading to more effective consolidation. On the other hand, if the immersion method was used to treat wood specimens, it seemed that penetration was easier, increasing the capacity of filling the open wood microstructure. Although these effects were already mentioned when talking about macroscopic mechanical properties, here they were more evident since the mechanical response came from the first hundreds of micrometers from the surface, which is the region of the sample where the fraction of penetrated polymer was higher. 

**Figure 10:**
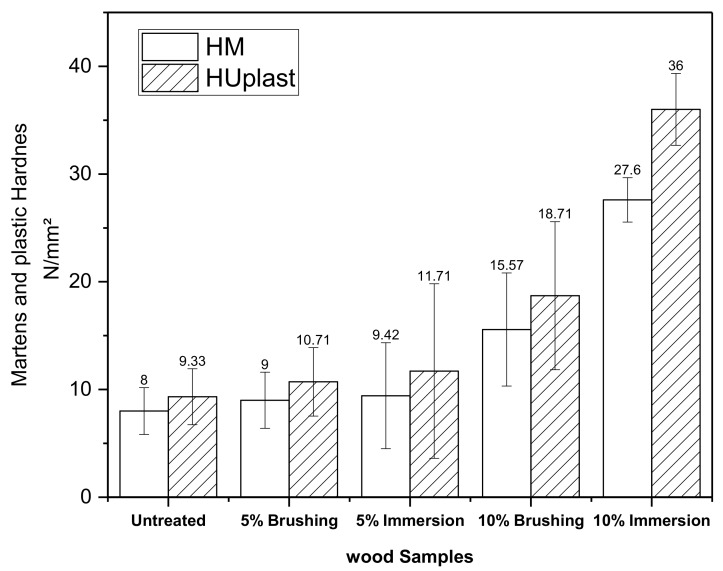
Martens hardness (HM) and plastic hardness (HU_plast_) for wood samples.

The Martens hardness (HM) and plastic hardness (HU_plast_) of wood samples obtained from the universal hardness tests are presented in Figure 10. Both Martens hardness (HM) and plastic hardness (HU_plast_), increased with PVBVA treatment following the same trend observed for the indentation depth. The hardness varied as follows: untreated ≈ 5%-brushing ≈ 5%-immersion < 10%-brushing < 10%-immersion. From these results, it was observed that samples treated with 5% solutions did not show significant changes in hardness. However, for samples treated with 10% PVBVA solutions, the greatest variations in hardness were obtained for the sample treated by immersion, as expected. Here, the same explanation given for the indentation results could be used.

**Figure 11:**
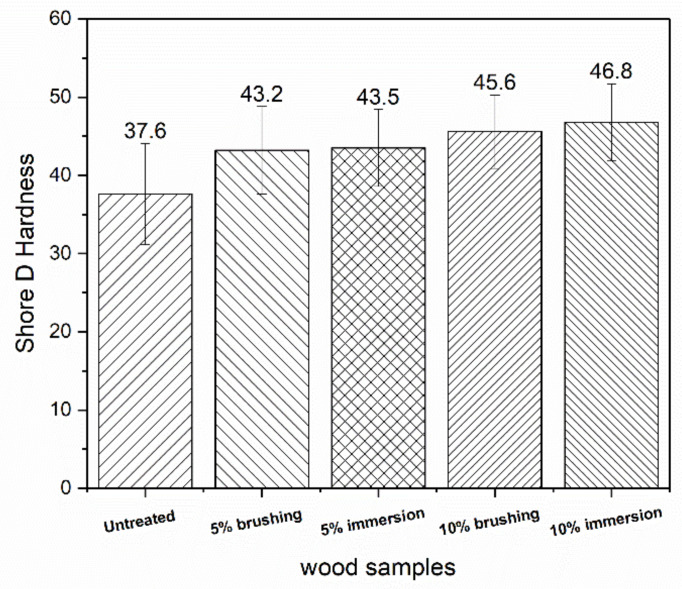
Average values of the Shore D hardness for the samples under study.

**Figure 12:**
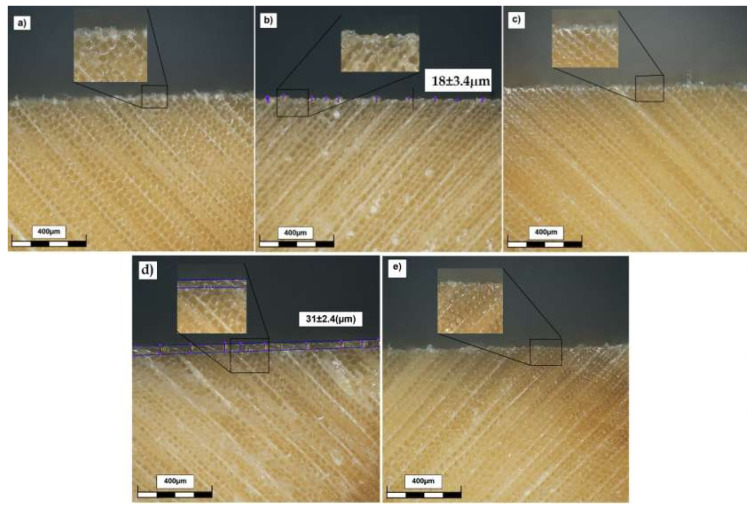
Cross-section micrographs obtained by optical profilometry to illustrate the cross-section profile of the wood samples: (**a**) untreated; (**b**) 5%-brushing; (**c**) 5%-immersion; (**d**) 10%-brushing; and (**e**) 10%-immersion.

The surface hardness of the samples was evaluated with the Shore D hardness test. In Figure 11, the average values of the Shore D hardness are shown. It can be observed that treatment of wood with PVBVA increased the hardness of the material. As expected, hardness increased significantly with the immersion in PVBVA solution treatment. Again, in terms of hardness, no significant differences were found as a function of the method used to apply the coating (brushing or immersion). The main difference was observed between treated and untreated wood. The results of the Shore D experiments obtained for untreated Fir wood were similar to those reported by other researchers [4]. Among the treated woods, the ones showing higher Shore D values were 10%-brushing and 10%-immersion, although the differences were, in general, within experimental variability. The fact that Shore D was not sensitive enough in this case to discriminate surface treatments in treated wood samples was because the penetration depth in the Shore D experiments was approximately 1 mm, thus providing a more global response of the material, whereas in the Martens hardness experiments, the penetration depth was close to ~150μm. This meant that the Martens hardness measurements might be a better option since, when used appropriately, they should allow us to discriminate better between these samples. It can be concluded that the coating slightly increased the surface hardness of the wood, which was important from the point of view of protection against environmental degradation, as for wood restoration purposes. To clarify the variations in surface properties, cross-section analysis of the surfaces was done (Figure 12), where it the thickness of the coatings obtained with the different treatments used in this study could be observed. 

As can be seen, when the brushing method was used to apply the polymer on the surface of the wood, a thin layer of polymer was clearly formed on the surface. Besides, as the concentration of the polymer solution increased, this layer became thicker, 18 μm when the 5% PVBVA solution was used and ~31 μm when the 10% PVBVA solution was used. When the wood samples were treated by immersion, a polymer layer was not visible at the magnification used (Figure 12); however, ATR-FTIR confirmed the presence of polymer at the surface. All these results, in fact, were in accordance with those obtained by ATR-FTIR, which pointed out that when brushing treatments were used, a higher amount (or thicker layer) of polymer was detected on the wood surface. One possible explanation of this observation could be associated with the high solvent evaporation rate when the brushing method was used to coat the wood. A rapid solvent evaporation together with a surface tension effect might lead to the formation of a polymer film over the wood voids, impeding subsequent entrance of polymer within the wood microstructure to exert the expected consolidation effect. 

## 4. Conclusions

Consolidation of Fir wood by treatments made with PVBVA was studied by surface and mechanical characterization. Two methods of application of the PVBVA solutions were considered, brushing and immersion, using two different concentrations, 5% and 10% (wt.%), in each case. FTIR spectroscopy evidenced the presence of the polymer on the treated surfaces and pointed out that when brushing treatments were used, a thicker layer of polymer was detected on the wood surface, confirmed by optical microscopy. One possible explanation was the high solvent evaporation rate when the brushing method was used to coat the wood, which may impede the penetration of the polymer into the wood microstructure to exert the expected consolidation effect. 

As a novel characterization, solution uptake experiments by means of dynamic contact angle measurements were done. The results seemed to indicate that there was a kind of induction time when the spreading process was no longer controlled by the viscosity of the solution, but by the interactions between the wood and the solution. Water contact angle (WCA) measurements indicated that wettability decreased after the treatment with the polymer, which is a good surface characteristic for conservation and restoration applications to prevent moisture absorption. To complete physico-chemical characterization of the surfaces, roughness measurements were done, but no clear differences were seen within the different surface treatments, at least at the scale considered using optical profilometry. 

From the point of view of mechanical characterization, the results obtained from the three-point bending test and Shore D experiments did not reveal any significant differences between the treated samples, which was interpreted considering that the overall response coming from the whole material was obtained with these experiments. The local mechanical properties measured at the surface revealed that the mechanical response of the surfaces, in terms of Martens hardness (HM), increased as follows: untreated < 5%-brushing ≈ 5%-immersion ≤ 10%-brushing < 10%-immersion. These results were correlated with optical cross-section profile observations, as well as with ATR-FTIR characterization of the samples. It could be concluded that from the point of view of surface mechanical characterization, HM measurements might be an interesting alternative to prove the effectiveness of a surface treatment on the consolidation of a wood artefact. 

As a general conclusion of this work, from the point of view of improving mechanical properties, the immersion method may seem as the most adequate one, both locally at the surface and in the bulk sample. In this work, it was found that the concentration of the solutions used to treat the wood played an important role, even more than the method of application. For this reason, when the immersion method might be difficult to use, for example in big artworks, one feasible approach might be to treat the surface of the samples with the 10% PVBVA solution by brushing. The 10%-brushing treatment had the second best local mechanical properties, and the general mechanical properties of the bulk sample increased, compared to the untreated wood. Besides, roughness was not modified significantly compared to the untreated wood, which may be the reason why there was not any variation in the visual appearance. Finally, the water contact angle increased, which indicated that this treatment may prevent moisture absorption and volumetric changes of the piece of wood.

## Figures and Tables

**Table 1:** Band assignment of FTIR absorption bands from PVBVA.

Wavenumber (cm^−^^1^)	Band Assignment
3300–4000	O–H stretching [40]
3100–2800	C–H stretching bands [41]
1738	C=O stretching [42]
1432–1345	CH_2_ bending vibration [43]
1377	symmetric bending CH_3_ [44] in-plane deformation of C–OH [39]
1133–1055	C–O–C–O–C stretching vibrations of cyclic acetal groups [43]
1241–991	C–O–C stretching vibration of acetate group [43]

**Table 2:** Roughness parameters Ra (μm) and Sa (μm) obtained by optical profilometry. The parameters were evaluated before and after the surface treatment for each sample.

Sample		Ra (μm)	Sa (μm)
5%-Brushing	Before	3.0 ± 1.0	4.2
	After	2.2 ± 0.5	2.7
10%-Brushing	Before	3.1 ± 0.6	3.6
	After	3.0 ± 1.0	3.9
5%-Immersion	Before	2.4 ± 0.8	3.2
	After	2.6 ± 0.5	3.2
10%-Immersion	Before	3.9 ± 0.8	5.4
	After	3.4 ± 0.9	4.3

**Table 3:** Mechanical parameters estimated from the three-point bending test, modulus of resistance (MOR) and modulus of elasticity (MOE).

Samples	MOR (MPa)	MOE (GPa)
Untreated	59 ± 5	5.0 ± 0.6
5%-Brushing	71 ± 9	6.1 ± 0.6
10%-Brushing	69 ± 10	6.0 ± 0.9
5%-Immersion	70 ± 8	6.3 ± 1.0
10%-Immersion	73 ± 9	6.2 ± 0.9

## Supplementary Materials

The following are available online at https://www.mdpi.com/2073-4360/12/5/1039/s1: Figure S1: Water contact angle (left) and representative images of water drops on the surface of different samples (right). Images correspond to: (a) untreated; b) 5%-brushing; (c) 10%-brushing; (d) 5%-immersion; and (e) 10%-immersion wood samples. Figure S2: Optical profilometry images corresponding to the sample treated with 5%-brushing PVBVA. (a) and (b) show the 2D and 3D images of the sample before treatment and (c) and (d) the corresponding ones upon the treatment with the 5% PVBVA solution at 555×; Figure S3: Optical profilometry images corresponding to the sample treated with 10%-brushing PVBVA. (a) and (b) show the 2D and 3D images of the sample before treatment and (c) and (d) the corresponding ones upon the treatment with the 10% PVBVA solution at 555×. Figure S4: Optical profilometry images corresponding to the sample treated with 10%-immersion PVBVA. (a) and (b) show the 2D and 3D images of the sample before treatment and (c) and (d) the corresponding ones upon the treatment with the 10% PVBVA solution at 555×. Figure S5: Force (kN) vs. displacement (mm) curves obtained in the three-point bending test experiments. Figure S6: Stress (MPa) vs. strain (%) curves obtained in the three-bending point test experiments.
