# Supplementary material for: Consolidation of Fir Wood by Poly(vinyl butyral-co-vinyl alcohol-co-vinyl acetate) Treatment: Study of Surface and Mechanical Characteristics
Source: Polymers (Basel). 2020 May 2;12(5):1039. doi: 10.3390/polym12051039 (PMC7284347; doi:10.3390/polym12051039)
Supplement: Supplementary file 1 [file polymers-12-01039-s001.pdf]

polymers

Article

Consolidation of Fir wood by Poly(vinyl butyral-co-vinyl alcohol-co-vinyl acetate) treatment: Study of surface and mechanical characteristics

Danial Harandi[1], Javier González-Benito[1], Dania Olmos[1,*]

[1] Dpto. Ciencia e Ingeniería de Materiales e Ingeniería Química, IQMAAB, Universidad Carlos III de Madrid Avda. Universidad, 15, 28911, Leganés, Madrid (SPAIN); dharandi@pa.uc3m.es; javid@ing.uc3m.es

* Correspondence: dolmos@ing.uc3m.es; Tel.: +34-916246048

Received: date; Accepted: date; Published: date

Supplementary Materials:

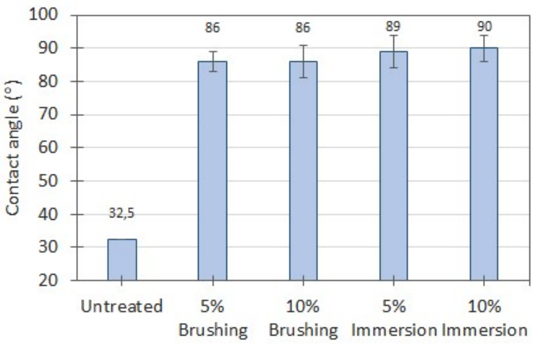

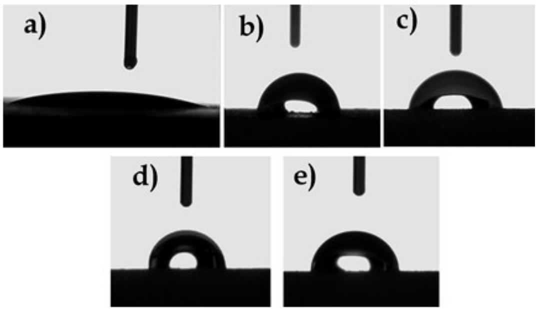

Figure S1. Water contact angle **(left)** and representative images of water drops on the surface of different samples (right). Images correspond to: (a) Untreated; b) 5%-Brushing; (c) 10%-Brushing; (d) 5%-Immersion and (e) 10%-Immersion wood samples.

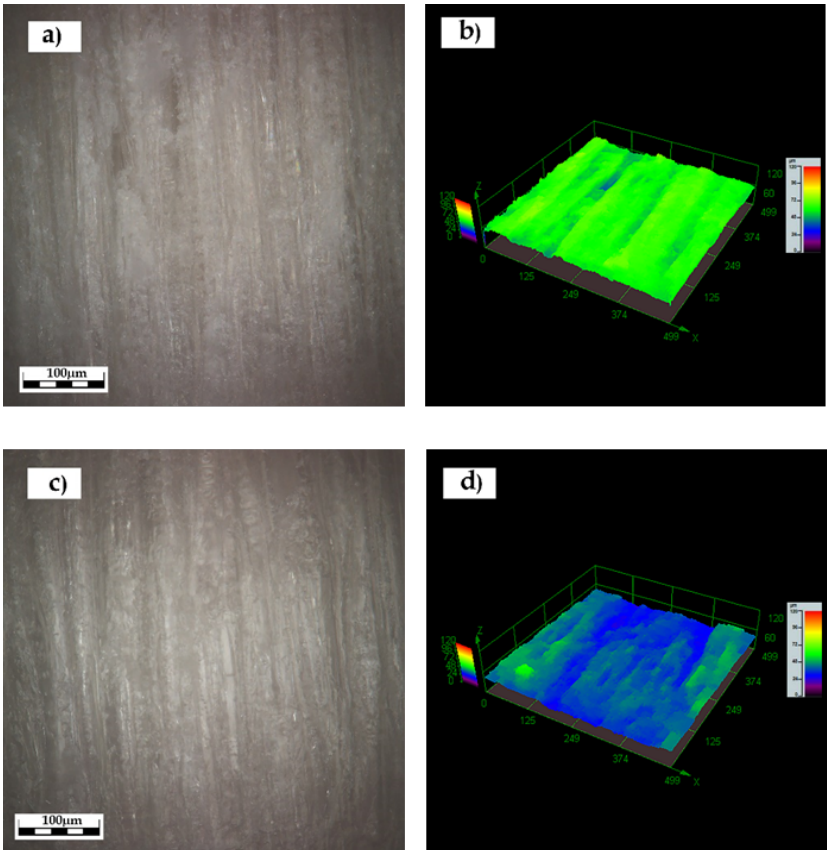

Figure S2: Optical profilometry images corresponding to the sample treated with 5%-Brushing PVBVA. (a) and (b) show the 2D and 3D images of the sample before treatment and (c) and (d) the corresponding ones upon the treatment with the 5% PVBVA solution at 555x.

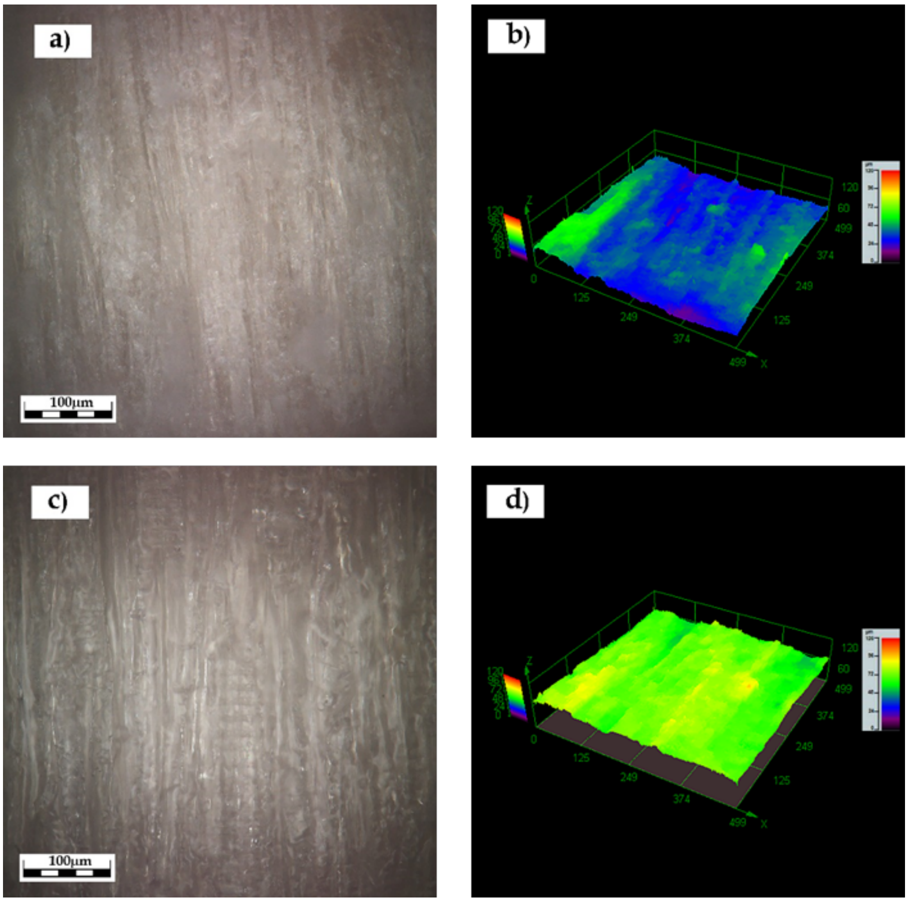

Figure S3: Optical profilometry images corresponding to the sample treated with 10%-Brushing PVBVA. (a) and (b) show the 2D and 3D images of the sample before treatment and (c) and (d) the corresponding ones upon the treatment with the 10% PVBVA solution at 555x.

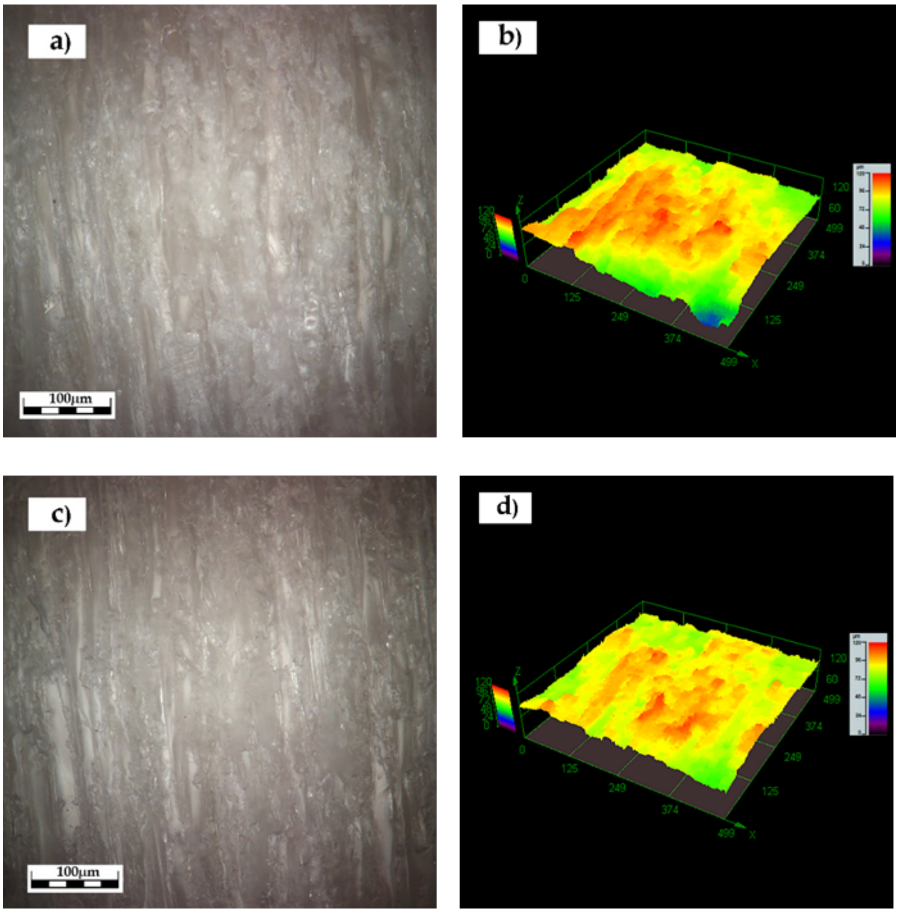

Figure S4: Optical profilometry images corresponding to the sample treated with 10%-Immersion PVBVA. (a) and (b) show the 2D and 3D images of the sample before treatment and (c) and (d) the corresponding ones upon the treatment with the 10% PVBVA solution at 555x.

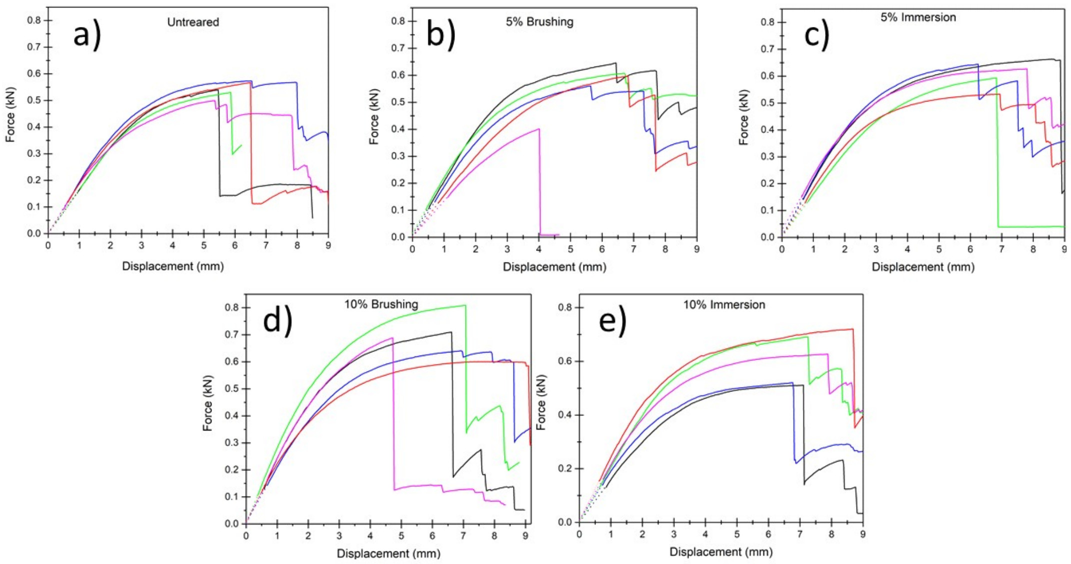

Figure S5: Force (kN) vs displacement (mm) curves obtained in the three-bending point test experiments.

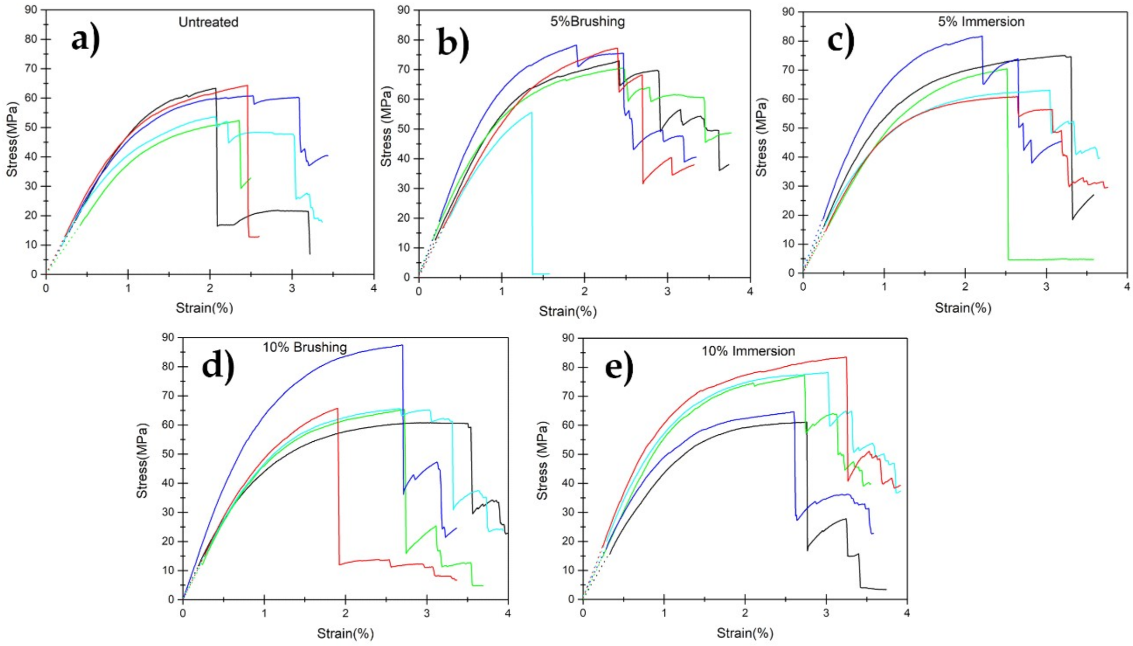

Figure S6: Stress (MPa) vs strain (%) curves obtained in the three-bending point test experiments.

© 2020 by the authors. Licensee MDPI, Basel, Switzerland. This article is an open access article distributed under the terms and conditions of the Creative Commons Attribution (CC BY) license (http://creativecommons.org/licenses/by/4.0/).
